# On automatic decipherment of lost ancient scripts relying on combinatorial optimisation and coupled simulated annealing

**DOI:** 10.3389/frai.2025.1581129

**Published:** 2025-05-30

**Authors:** Fabio Tamburini

**Affiliations:** Department of Classical Philology and Italian Studies, University of Bologna, Bologna, Italy

**Keywords:** ancient script decipherment, combinatorial optimization, k-permutations, coupled simulated annealing, evaluation benchmarks

## Abstract

This paper introduces a novel method for addressing the challenge of deciphering ancient scripts. The approach relies on combinatorial optimisation along with coupled simulated annealing, an advanced technique for non-convex optimisation. Encoding solutions through k-permutations facilitates the representation of null, one-to-many, and many-to-one mappings between signs. In comparison to current state-of-the-art systems evaluated on established benchmarks from literature and three new benchmarks introduced in this study, the proposed system demonstrates superior performance in enhancing cognate identification results.

## 1 Introduction

Numerous ancient scripts around the world remain undeciphered, with many of them dating back millennia. The challenges in deciphering these scripts stem from factors such as insufficient inscriptions, the absence of known language descendants utilizing these scripts, and uncertainty about whether the symbols truly form a writing system.

In the Aegean region, for instance, three syllabic scripts — Linear A, Cretan Hieroglyphs, and the Cypro-Minoan script — despite their historical interconnectedness, have resisted decryption efforts. While this study addresses general decipherment challenges, its primary focus lies in investigating undeciphered scripts from the eastern Mediterranean during the Bronze Age or early Iron Age.

Unraveling an ancient script is generally a highly intricate task, often necessitating the division of the challenge into distinct subproblems. This approach serves to derive specific answers or simplify the task by breaking it down into more manageable components. In literature, numerous contributions address these subproblems, offering computational methods tailored to each, frequently focusing on a particular script. The sequential tasks typically involve: (a) determining if a set of symbols genuinely constitutes a writing system, followed by (b) devising procedures to segment the symbol stream into individual signs. Subsequently, (c) reducing the set of signs to the minimal collection for the given writing system, thereby forming the alphabet (or syllabary, or sign inventory), and identifying all allographs. Once this minimal yet comprehensive symbol set is established, the process involves (d) assigning phonetic values and, ultimately, (e) attempting to align phonetic transcriptions with a specific language.

The subsequent sections provide an in-depth exploration of the computational perspective regarding the five points mentioned earlier.

### 1.1 Pictures or language?

When presented with symbols etched onto stones or inscribed on tablets and other mediums, one of the initial tasks involves determining if these symbols signify a form of language or another means of communication not linked to a natural language.

In this context, two primary lines of computational studies have tackled this issue nearly simultaneously. Rao et al. (2009, 2010) conducted an analysis of the undeciphered Indus Valley script to establish whether it indeed represents a natural language. The authors provide supporting evidence for the linguistic hypothesis by demonstrating that the script's conditional entropy aligns more closely with that of natural languages than with various types of non-linguistic systems.

Around the same period, Lee et al. (2010) employed a two-parameter decision-tree technique capable of discerning the nature of communication within very small corpora. When applied to a collection of a 100 stones intricately carved by the Picts, an Iron Age culture in Scotland, featuring stylised symbols, the study concluded that these symbols did not exhibit randomness or sematographic (heraldic) characteristics. Instead, they displayed attributes indicative of a written language.

Regrettably, achieving a consensus on such approaches remains elusive. A study by Sproat (2010) strongly critiques this method, utilizing a more extensive set of non-linguistic and comparative linguistic corpora than those employed in previous studies. The study demonstrates that none of the previously proposed methods are reliably effective in decisively determining whether the considered symbols truly represent a writing system. Simultaneously, it introduces a novel measure based on repetition that classifies them as non-linguistic, contradicting the conclusions of earlier works.

### 1.2 Script segmentation

A major hurdle in deciphering undeciphered scripts lies in the segmentation of words and signs. Identifying these two fundamental units is essential before commencing the decipherment process, whether through manual efforts or with the assistance of computational techniques. This challenge is also evident when endeavoring to construct electronic corpora for undeciphered scripts, a crucial initial step in computational epigraphy. The preparation of these corpora from raw archaeological data demands substantial human effort.

Palaniappan and Adhikari (2017) introduced an automated tool leveraging machine learning algorithms to assist in epigraphical research. This tool presents a deep learning pipeline designed to take input images of the undeciphered Indus script and generate, as output, a string of graphemes suitable for integration into a standard corpus. The process involves initial decomposition of the input image into regions and subsequent classification using a convolutional neural network to distinguish textual and/or graphical elements. This network adeptly classifies the graphemes with remarkable accuracy, underscoring the substantial promise of employing deep learning methodologies in the realm of computational epigraphy.

Furthermore, Luo et al. (2021) introduces a comprehensive approach that simultaneously addresses word segmentation and cognate alignment. This method utilizes phonological constraints within a generative stochastic model and includes a novel technique for discerning closely related languages.

As an illustration, examining Rongorongo, a script potentially documenting the local Rapanui language on Easter Island, poses challenges. The segmentation of this script into linguistic units—be they sounds, syllables, or morphemes—remains unclear. Additionally, various small shapes, nearly identical, intricately combine in different configurations to create complex signs (Davletshin, 2012; Valério et al., 2022).

### 1.3 Building a uniform set of signs

Upon successfully devising a method to segment the script into meaningful linguistic units, scholars encounter the initial challenge of identifying a sign-list. This task proves intricate due to variations introduced by scribe writing styles and the evolution of symbols over time, complicating the identification and management of allographs.

In addressing this challenge, Skelton (2008) and Skelton and Firth (2016) applied phylogenetic systematics to the realm of writing systems. Their focus was particularly on Linear B, a pre-alphabetic Greek script. Through this method, they scrutinized the evolution of the Linear B script over time, taking into account scribal hands as an additional source of variation. This application showcased the efficacy of phylogenetic analysis in understanding the development of writing systems.

Born et al. (2019) and Born et al. (2023b) employed computational linguistics techniques to analyse Proto-Elamite, an ancient script from the 3rd millennium BC used across the Iranian plateau. Their approach involved utilizing three distinct clustering algorithms to create and explore sign groups based on their occurrences and co-occurrences within texts.

Corazza et al. (2022) delved into the analysis of the Cypro-Minoan syllabary. Their methodology aimed to scrutinize the tripartite division (CM1, CM2, CM3) of Cypro-Minoan, evaluating its consistency through a multi-disciplinary approach. This encompassed considerations related to paleography and epigraphy, along with the application of deep learning-based strategies. Using an unsupervised state-of-the-art convolutional neural model without prior knowledge of the script, they found that the use of different media significantly influences the uniformity of sign shapes and highlighted graphic proximity among signs inscribed on similar supports. Notably, their results consistently supported the validation of a unitary, single Cypro-Minoan script, countering the current literature's discussion of a division into three subgroups. This conclusion suggests that most sign differences arise from the choice of epigraphic supports, providing insights into the rationalization of the sign inventory proposed by Olivier (2007) and suggesting potential sign mergers.

### 1.4 Assigning signs values (phonetic/numeric)

This represents a crucial phase in the decipherment process. However, as discussed in the following section, most literature works tend to address this step concurrently with the challenge of identifying the language associated with the examined script.

A noteworthy exception is found in the work of Corazza et al. (2021), who utilized computational techniques — primarily constraint programming and optimisation methods — to assign for the fraction symbols present in Linear A. Minoan Linear A, an undeciphered script prevalent for administrative purposes in Bronze Age Crete, poses challenges regarding the precise mathematical values of its numerical fractions. Building upon previous analyses that proposed hypothetical values for certain fractions, they expanded their investigation to assess values for more challenging cases. The results, derived from meticulous palaeographical analysis and employing computational, statistical, and typological strategies, revealed a significant convergence. They pointed toward a systematic assignment of mathematical values for the fraction signs in Linear A.

A comparable study by Born et al. (2023a) addresses the task of disambiguating between various numerical interpretations in Proto-Elamite, aiming to determine the values of numeric quantities documented in ancient texts. The authors algorithmically extracted a list of potential readings for each numeral notation and proposed disambiguation techniques based on structural properties of the original texts and classifiers trained using the bootstrapping algorithm.

### 1.5 Define signs values and match sign sequences with a known language

Every contemporary endeavor to decrypt ancient scripts using computational tools relies on contrasting a missing script or language wordlist with words from a deciphered and known language. These computational methods need to address two main challenges:

- The initial challenge arises when there is a potential lack of alignment between the two scripts; in such instances, the phonological values of the lost symbols may remain unidentified, requiring a preliminary matching between the scripts before aligning the two wordlists;
- The second challenge entails finding a way to match the two wordlists by identifying “cognate” words^1^.

Certain scholarly works focus exclusively on detecting cognates within the same script (Bouchard-Côté et al., 2009) or directly utilizing sound representations from the International Phonetic Alphabet (Hall and Klein, 2010). Notably, these studies primarily involve languages that share strong typological similarities.

In contrast, the most sophisticated studies presented in the last years on the automatic decipherment of lost languages propose systems that generate both sign mappings between different scripts and mappings of words into their corresponding cognates (e.g., Snyder et al., 2010; Berg-Kirkpatrick and Klein, 2011; Luo et al., 2019, 2021). These studies adopt a shared computational approach, structuring the algorithm as a two-step procedure inspired by the Expectation–Maximization (EM) algorithm — an iterative method for finding (local) maxima or minima. The initial step suggests a provisional working match between the two “alphabets”^2^. The subsequent step, building upon the established script match, endeavors to align the two word lists by proposing potential cognates. Initially, the script matching and cognate matching may be nearly random, but through multiple iterations, the process is expected to converge, offering both a script match and a list of potential cognates. The crucial aspect revolves around identifying a suitable function, optimized through this iterative process, that effectively captures the concept of word matching while incorporating linguistic constraints related to scripts, words, and potentially sounds. The following section provides an overview of the most pertinent analyses, in the authors' perspective, that address the decipherment problem in an automated manner, all adhering to the general scheme just discussed.

The pioneering work by Snyder et al. (2010) marked the initiation of the modern approach to the computational decipherment problem. Their approach depends on utilizing a non-parallel corpus in a recognized, closely related language, producing both alphabetical mappings and translations of words into their corresponding cognates. Their work, based a non-parametric Bayesian framework, captures both low-level character mappings and high-level correspondences at morphological level. They tested this approach comparing Ugaritic with Old Hebrew obtaining promising results: the model accurately mapped 29 of 30 signs to their Old Hebrew counterparts and deduced the correct cognate for 60% of Ugaritic words. Unfortunately, the code for this method is not available.

In a distinct approach, Berg-Kirkpatrick and Klein (2011) created a straightforward objective function that, when optimized, yields precise solutions for both decipherment and the identification of cognate pairs. The proposed solution, characterized by its simplicity and elegance, employs binary variables to control the alignment between symbols in the two scripts and the correspondence between the two lexicons. Relying solely on an integer combinatorial optimisation procedure, their system exhibited efficacy in solving the identical problem presented by Snyder et al. (2010) and on a new task involving romance languages. While the code for this method is unfortunately unavailable, replicating the approach appears feasible as it is clearly described in the paper.

Luo et al. (2019) introduce a sophisticated neural approach that, in our assessment, stands out as one of the most promising methods for the automatic decipherment of lost languages. Addressing the challenge of limited supervision information, their model incorporates known language change patterns documented by historical linguistics. Sign mapping is executed by a bidirectional recurrent neural network, and the cognate matching procedure is formalized as a minimum-cost flow problem. The method was applied to the benchmark problem posed by Snyder et al. (2010) and to a novel dataset encompassing Linear B and ancient Greek lexica, yielding highly favorable mapping results. Notably, the code and datasets necessary to reproduce their results are made available to the community.

In a subsequent work, Luo et al. (2021) addressed a more complex task dealing with scripts that were not completely segmented into words and situations where the closest known language was unknown. By utilizing extensive linguistic constraints that mirror consistent patterns in historical sound change, the authors captured the natural phonetic structure by acquiring phone embeddings based on the International Phonetic Alphabet. The resulting generative framework concurrently incorporates models for both word segmentation and cognate alignment, guided by phonetic and phonological constraints. They tested their method on deciphered languages, namely Gothic and Ugaritic, as well as an undeciphered language, Iberian, demonstrating that incorporating phonetic geometry yields clear and consistent improvements. Additionally, the authors introduced a measure for language closeness, correctly identifying related languages for Gothic and Ugaritic. The code and data for their work are made accessible to the community.

### 1.6 Other computational tools

Epigraphy, the study of inscriptions, plays a crucial role in extracting evidence related to the thoughts, language, society, and history of past civilisations. However, many inscriptions have suffered damage over time. The endeavor to restore these invaluable sources, to the extent possible, holds the potential to provide additional information that can enhance and deepen our understanding of a particular population.

A notable contribution in this realm comes from Assael et al. (2022), who introduce Ithaca, a deep neural network specifically crafted for restoring text, determining geographical origins, and assigning chronological attributes to ancient Greek inscriptions. Ithaca is specifically crafted to support historians in their work, demonstrating its capability to enhance accuracy in reading and attributing inscriptions.

Similarly, Fetaya et al. (2020) present a method that employs recurrent neural networks to model the language inscribed on clay cuneiform tablets. This approach aims to assist scholars in reconstructing fragmented sections of ancient Akkadian texts from the Achaemenid period in Babylonia.

An important factor influencing the interpretation of ancient writing systems is the inherent variation introduced by different scribal hands. Paleography faces the challenge of identifying the authorship or distinguishing differences when the writing style varies.

In a study by Srivatsan et al. (2021), neural feature extraction tools were employed to analyse scribal hands in the Linear B writing system. Their system assigns a shared vector embedding to each sign written by the same scribal hand, representing the author's stylistic patterns. Additionally, signs representing the same syllable share a vector embedding that captures the identifying shape of the character.

Similarly, a study by Popović et al. (2021) focused on the Great Isaiah Scroll, one of the Dead Sea Scrolls. By employing pattern recognition and artificial intelligence techniques, the research revealed that two main scribes, each exhibiting distinct writing patterns, were responsible for inscribing the scroll. This finding contributes new insights into the ancient scribal culture of biblical texts, indicating that ancient biblical manuscripts were not exclusively copied by a single scribe.

Finally, in the work by Lastilla (2022), evidence is presented that automatic techniques, specifically self-supervised learning applied to convolutional neural networks, can effectively address the challenge of handwriting identification for medieval and modern manuscripts. This emphasizes the strong capabilities of self-supervised methods in digital paleography, particularly in scenarios where unlabelled data is prevalent and generating labeled data poses difficulties.

## 2 Materials and methods

The primary benchmarks for our proposal are the studies conducted by Berg-Kirkpatrick and Klein (2011) and Luo et al. (2019).

In their work, Berg-Kirkpatrick and Klein (2011) introduced an approach that serves as an inspiration for our work, emphasizing the potential of addressing the decipherment problem as a pure function optimisation problem. However, their results no longer represent the state-of-the-art, as subsequent works have surpassed them.

Conversely, the study by Luo et al. (2019) demonstrates a system capable of achieving commendable results, although it lacks the flexibility required for our purposes. In this system, a recurrent Neural Network (NN) is employed to establish the mapping between lost and known signs, despite the advantage of using contextual information to perform the task, it lacks the adaptability necessary for addressing two practical decipherment challenges. Firstly, paleographers often possess partial knowledge about the mapping of certain signs, and this information needs to be incorporated into the system. Secondly, real inscriptions are frequently broken or damaged, leading to unreadable signs, requiring the incorporation of uncertainty into the system, potentially through the use of wildcards or other special symbols. Implementing such treatments proves challenging in a recurrent NN. Additionally, deep NNs typically demand substantial data for effective training, a condition not always met in real-world situations. As mentioned earlier, our focus is on examining undeciphered scripts from the Aegean region, necessitating a more adaptable system capable of accommodating partial readings, incorporating fixed knowledge, and operating effectively with limited data.

Inspired by the work in Berg-Kirkpatrick and Klein (2011), we will introduce a flexible encoding of potential solutions and an “energy function" designed to assess the quality of a given solution. This assessment considers both signs matching and lexica matchings. By minimizing the energy function, our goal is to explore viable solutions to a decipherment problem.

To facilitate discussions in the following sections, we introduce some notation. *L*_*s*_ and *K*_*s*_ represent two linearly ordered sets (a set with a total order) containing respectively the signs in the lost and known languages, respectively. The cardinalities of these sets are denoted as |*L*_*s*_| and |*K*_*s*_|, with *l*^*i*^ and *k*^*j*^ representing the i-th and j-th elements in the ordered sets. Additionally, *L*_*lex*_ and *K*_*lex*_ represent the two lexica, with |*L*_*lex*_| and |*K*_*lex*_| denoting their respective numbers of words.

### 2.1 Solution coding

The fundamental tool for encoding a solution to the problem is the *k*-permutation without repetition. Consider *n* objects denoted as *p*_1_, …, *p*_*n*_. Let *s*_1_, …, *s*_*k*_ represent *k* slots (*k* ≤ *n*), where *k* objects can be assigned. A *k*-permutation of *n* objects refers to one of the possible ways to select *k* objects and place them into the *k* slots. Each object can only be chosen once and the objects order matters. The number of possible *k*-permutations is given by $P_{n,k}=\frac{n!}{\left(n-k\right)!}$. For this work, we consider the *k*-permutation of the first *n* integer numbers.

To identify an appropriate sign assignment between the lost language and the known language, a generic solution σ should have the flexibility to represent multiple assignments in both directions, while being mindful of the combinatorial explosion issue.

Let's consider the scenario where |*L*_*s*_| ≤ |*K*_*s*_|. In this case, some lost signs must be mapped to more than one known sign. This situation can be efficiently encoded using a single *k*-permutation σ with *n* = *N*·|*L*_*s*_| and *k* = |*K*_*s*_|, where *N* = 2, 3, .... Each known sign *k*_*j*_, positioned at *j* ≤ *k* in the *k*-permutation σ = 〈σ_1_, ..., σ_*k*_, ..., σ_*n*_〉, is then mapped to a set of lost signs through the function $MapS^{\sigma }:K_{s}\to P\left(L_{s}\right)$,


$$MapS^{\sigma }\left(k_{j}\right)=l_{\sigma_{j}\;\mathrm{mod}\;|L_{s}|}$$

where $P\left(L_{s}\right)$ is the power set of *L*_*s*_.

In the alternate scenario where |*L*_*s*_|>|*K*_*s*_|, we can define a solution σ comprising *M* *k*-permutations, where *M* = 2, 3, ..., concatenated successively. Each permutation is handled in the same manner as described earlier, but now *N* can also be equal to 1.

By structuring the possible solutions σ in this manner, every symbol in the lost language has the capacity to be assigned from 0 to a maximum of *N*×*M* potential assignments of known signs, offering a considerable degree of flexibility in signs matching. Given that, in the definition of *k*-permutations the parameter *N* governs the well-formedness of the fundamental structure supporting solution definition. It ensures that every known sign is assigned to at least one lost sign, addressing the various situations that arise when |*L*_*s*_| ≤ |*K*_*s*_|. Additionally, *M* dictates how many times a known sign will be assigned to a lost sign. *N* and *M* are not independent parameters as they interact in a complex manner to regulate the number of multiple assignments in both directions.

**Figure 1:**
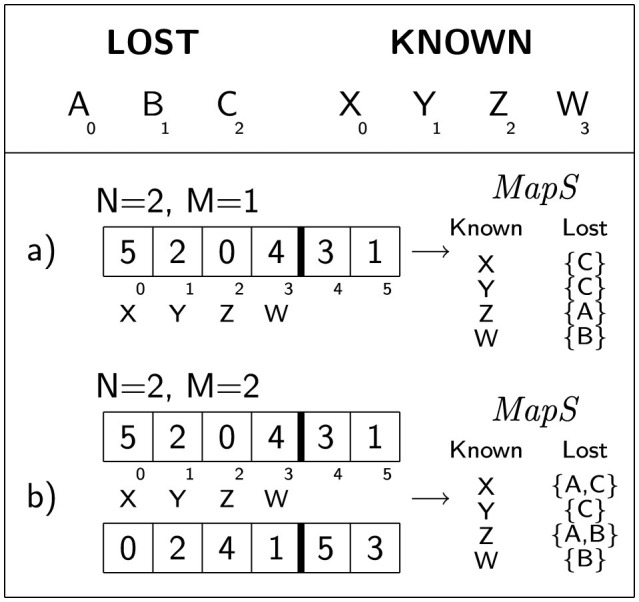
Here are two straightforward examples of solution coding: **(a)** When *M* = 1, the initial |*K*_*s*_| cells encode the mapping *MapS* for the known signs to the lost signs (depicted on the right). Using k-permutations of size *N*·|*L*_*s*_| accommodates one-to-many mappings from lost to known signs (refer to the definition of *MapS*). **(b)** When *M* = 2, two k-permutations are employed, allowing for one-to-many assignments from known to lost signs. In both cases, there is a possibility that a lost sign may not receive any assignment (not illustrated in the picture).

Figure 1 illustrates two concise examples of the suggested schema for encoding solutions.

An additional advantage of *k*-permutations regards the facts that it exists an isomorphism between *k*-permutations and natural numbers (Patel, 2022). Consequently, each solution encoded using our schema can be translated into *M* integers, and, for practical problems with *M* ≤ 2, fragments of the search space can be inspected using a 2D/3D graph.

### 2.2 Energy function

The second essential component in the proposed approach involves devising a suitable energy function capable of assessing the quality of a provided solution for a decipherment problem.

As previously mentioned, Luo et al. (2019) divided the optimisation process into two distinct, iteratively repeated steps. The first step calculates the optimal match between signs given a lexicon match, and once the signs match is fixed, the second step determines the best match between lexica. In contrast, we opted for a different approach, formulating an energy function that evaluates the quality of both aspects simultaneously.

#### 2.2.1 Lost words expansion and transliteration

To transliterate the lost lexicon, it is necessary to define the inverse function of *MapS*, denoted as $invMapS^{\sigma }:L_{s}\to P\left(K_{s}\right)$, which assigns each lost sign to the set of known signs mapped to it, as


$$invMapS^{\sigma }\left(l_{i}\right)=\left\{k_{j}|l_{i}\in MapS^{\sigma }\left(k_{j}\right)\right\}\;.$$

Expanding upon this definition, we can introduce the transliteration and expansion function *TrExp*^σ^ for a given lost word *lW* = 〈*lW*_1_, ..., *lW*_*n*_〉, with *lW*_1_, ..., *lW*_*n*_ the sequence of signs forming *lW*, as


$$TrExp^{\sigma }\left(lW\right)=\left\{tW|tW=\langle q_{1},...,q_{n}\rangle ,q_{j}\in invMapS^{\sigma }\left(lW_{j}\right)\right\}.$$

*TrExp* transliterates each lost word into the known alphabet and links it to a set of transliterated words formed by any combination of known signs permitted by the mapping *invMapS*. While this approach could potentially result in a combinatorial explosion, the fact that *N* and *M* are typically very small integers (almost always ≤ 3) mitigates the severity of this issue. Table 1 provides an example of this process.

**Table 1:** Example of transliteration and expansion using the same sets of signs as depicted in Figure 1b.

***l*_*i*_**	** $invMapS^{\sigma }\left(l_{i}\right)$ **	***lW***	***TrExp*^σ^(*lW*)**
A	{Z,X}	AA	{ZZ,ZX,XZ,XX}
B	{W,Z}	BC	{WX,WY,ZX,ZY}
C	{X,Y}	ABC	{ZWX,ZWY,ZZX,ZZY,

#### 2.2.2 Word matching

A conventional method for comparing strings involves the use of the so-called *edit distance* (ED), also known as Levenshtein distance. We employed this measure to compare the expanded transliterations of lost words to known words. The standard ED definition counts the number of sign insertions, deletions, and substitutions required to transform the first string into the second. We adapted this definition, following the ideas presented in Wang et al. (2021), to incorporate two wildcards that can be particularly useful in real settings. In actual inscriptions, signs are often damaged or indistinguishable; in such situations, it might be preferable to process this data while acknowledging the reading challenges. For this purpose, we introduced the special sign “?” to indicate a single unreadable sign and “*” to indicate multiple unreadable signs, both allowed only in lost words.

**Figure 2:**
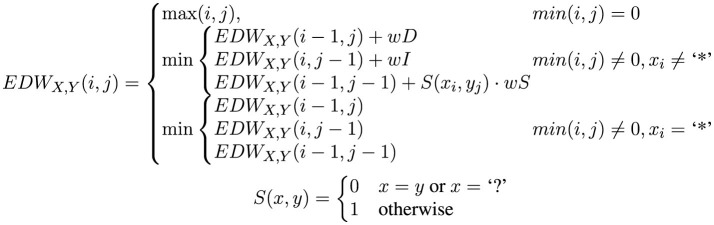
Definition of the edit distance with Wildcards: *wD*, *wI*, and *wS* represent the weight penalties for sign deletion, insertion, and substitution, respectively. For this study, all these weights have been set to 1. “?” and “*” are the two wildcards considered in this study.

Consider two words to be compared, *X* = 〈*x*_1_, ...*x*_*n*_〉 and *Y* = 〈*y*_1_, ...*y*_*m*_〉, with *n* and *m* being their respective lengths. The ED with wildcards used in this study, denoted as *EDW*_*X, Y*_(*n, m*), is defined as shown in Figure 2.

The general edit distance, including our variation with wildcards, does not consider word lengths and is not ideal for comparing the distance between sets of words. Consequently, many studies have introduced a form of normalization for edit distance values. Recognizing the valuable properties (Fisman et al., 2022) of the *Generalized Edit Distance* proposed by Li and Liu (2007)^3^, we normalized *EDW* as


$${\bar{EDW}}_{X,Y}=\frac{2\cdot EDW_{X,Y}}{|X|+|Y|+EDW_{X,Y}}$$

where |·| represents the word length.

We utilized $\bar{EDW}$ to compare the transliterated and expanded lost lexicon, generated by applying the *TrExp* function to each word in *L*_*lex*_, against the known words in *K*_*lex*_ (as discussed in the next section). We implemented the $\bar{EDW}$ function in an efficient code that is compatible with GPUs^4^.

#### 2.2.3 Lexica matching

Cognacy relations within the two examined language lexicons may involve 1-to-many, many-to-1, or many-to-many mappings between cognate words. To appropriately address these possibilities and facilitate accurate evaluation, we introduced a specialized variant of the standard Linear Sum Assignment (LSA) problem, also known as the Hungarian algorithm, for lexica matching. Instead of matching individual words, our method involves matching groups of words on both the lost and known sides. On the lost language side, this accommodates different transliterations of the same lost word resulting from multiple assignments to the same lost sign (as defined by functions *invMapS* and *TrExp*). On the known language side, it considers sets of possible cognates associated with the lost word(s) in a given benchmark dataset.

To introduce our modified version of the LSA algorithm, let's establish a partition $K_{lexG}=K_{lexG}^{1},...,K_{lexG}^{G}$ of *K*_*lex*_, where $K_{lexG}^{j}$ denotes a set of known cognates in the dataset. Subsequently, we can define the variables *A*_*i, j*_∈0, 1 to represent the lexica alignment obtained by the LSA algorithm (with *A*_*i, j*_ = 1 if and only if *lW*^*i*^ is assigned to $K_{lexG}^{j}$). The LSA problem to be solved can then be expressed as


$$\min\overset{\left|L_{lex}\right|}{\underset{i=1}{\sum }}\overset{\left|K_{lexG}\right|}{\underset{j=1}{\sum }}A_{i,j}\cdot \left[\underset{\begin{array}{c} X\in TrExp^{\sigma }\left(lW^{i}\right)\\ Y\in \overset{j}{\underset{lexG}{K}} \end{array}}{\min}{\bar{EDW}}_{X,Y}\right]$$

$$\begin{array}{c} \;\text{s.t.}\underset{i}{\sum }A_{i,j}=1,\;j=1,2,...,|K_{lexG}|\\ \;\underset{j}{\sum }A_{i,j}=1,\;i=1,2,...,|L_{lex}| \end{array}$$

and, after solving the LSA and determining the values for the matching variables *A*, the Energy function *E* for a given problem solution σ can be defined as


(1) $$\begin{array}{c} E\left(\sigma \right)=\overset{\left|L_{lex}\right|}{\underset{i=1}{\sum }}\overset{\left|K_{lexG}\right|}{\underset{j=1}{\sum }}A_{i,j}\cdot \left[\underset{\begin{array}{c} X\in TrExp^{\sigma }\left(lW^{i}\right)\\ Y\in K_{lexG}^{j} \end{array}}{\min}{\bar{EDW}}_{X,Y}\right] \end{array}$$

See Table 2 for a simple example of the lexica matching process.

**Table 2:** An illustrative instance of the lexica matching process is presented here.


*L*_*lex*_		*K*_*lex*_					
*lW*	*TrExp*	**WX**	**WW**	**XX**	**XWY**	**XWZ**	
AA	ZZ	2	2	2	3	2	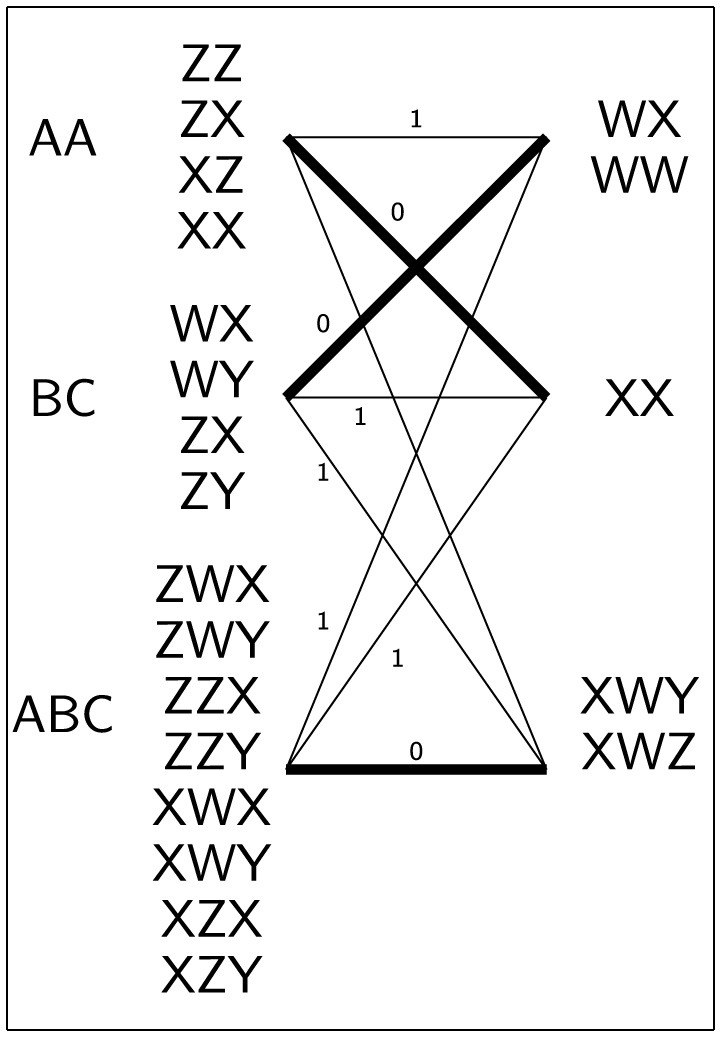
BC	WX	0	1	1	2	2		
ABC	ZWX	1	2	2	2	2		

The lost lexicon mirrors the example in Table 1, while the known lexicon comprises five words organized into three sets of cognates. On the left side, the cost matrix is generated using the edit distance (normalized version not utilized for clarity). The values enclosed in a box highlight the minimum costs considering the respective groups. On the right side, these minimal values represent the costs for an LSA problem, determining the minimum-cost matching (indicated by thick lines) between the two lexica while adhering to the groupings in the lost and known lexica.

It is noteworthy that the calculation of the energy function *E* for a specific solution σ is inherently derived from the solution. This process involves translating the solution coding into sign assignments using the function *TrExp* and subsequently aligning the two lexica through the LSA procedure outlined above.

#### 2.2.4 Penalty factors

To regulate the entire process and facilitate the optimisation procedure in discovering reliable solutions, we introduced regularization factors into the energy function *E*. Given that our method relies on a flexible assignment schema, permitting no assignments to lost signs and multiple assignments of known signs, it is essential to ensure that the optimisation procedure does not overuse these options. Generally, having no assignments to lost signs rarely leads to a satisfactory solution, and exaggerating with multiple assignments of known signs can be detrimental. To discourage solutions with these characteristics, we incorporated two penalisation factors. If we denote *#UA*(σ) as the number of lost signs without any assignment and *#MA*(σ) as the number of known signs with multiple assignments for a given solution σ, the final energy function to be minimized is


(2) $$\begin{array}{c} E^{\prime }\left(\sigma \right)=E\left(\sigma \right)+\lambda \cdot \left[\#UA\left(\sigma \right)+\#MA\left(\sigma \right)\right] \end{array}$$

where the parameter λ allows to weight the contribution of penalisation factors on the energy function.

### 2.3 Energy optimisation using coupled simulated annealing

Structuring our problem as a comprehensive global optimisation process has guided us to minimize the energy function *E*′, as defined earlier, employing various metaheuristic techniques found in the literature, such as tabu-search, genetic and evolutionary methods, ant colony optimisation, simulated annealing, and others.

The Coupled Simulated Annealing (CSA) method, introduced by de Souza et al. (2010), is a global optimisation technique based on Simulated Annealing (SA). CSA involves a set of parallel SA processes (with *#Anns* denoting the number of annealers), interconnected by their acceptance probabilities. The coupling mechanism incorporates a term in the acceptance probability function that relies on the energies of the ongoing states of all SA processes, fostering cooperative behavior through information exchange among parallel annealing processes. Furthermore, the coupling aspect offers insights that can guide the overall optimisation process toward the global optimum. The original authors present a system capable of utilizing the acceptance temperature to regulate the variance of acceptance probabilities through a straightforward control scheme (referred to as “CSA-MwVC” in the original work). This contributes to enhanced optimisation efficiency by mitigating the algorithm's sensitivity to initialization parameters while steering the optimisation process toward quasi-optimal states.

After experimenting with various techniques, we opted for CSA for two primary reasons: (a) it offers easy parallelisation on a multicore CPU, facilitating highly parallel computations with minimal information exchange, and (b) its inherent control mechanism over the variance of acceptance probabilities autonomously manages the annealing process, eliminating the need for intricate annealing schemes often requiring tuning for specific problems and datasets.

For the implementation of CSA we relied on a code specifically tailored for permutation-based problems^5^, configured to incorporate 16 parallel annealers.

The generic SA algorithm is straightforward: given a solution, we must perturb it to obtain a new solution in its neighborhood, which is then accepted or rejected based on a stochastic decision influenced by the new solution's energy and the current global system temperature. The selection of a neighboring solution involves a critical step to ensure a proper sampling of the solution space. Fortunately, a comprehensive study by Tian et al. (1999) examined the most promising “moves” for solutions based on permutations, with the swapping of two items in the permutation deemed the most effective move for assignment problems. To prevent the system from becoming trapped in a local minimum, we also introduced a random *p*-swap perturbation with a probability of 0.1, where *p* decreases with the generation temperature governed by the CSA schedule.

Concerning the stopping criterion for the CSA process, we opted to conclude the annealing after 100 temperature updates without observing any improvement in the best solution (*best*_σ).

Refer to Algorithm 1 for an overall overview of the entire optimisation process.

**Algorithm 1:**
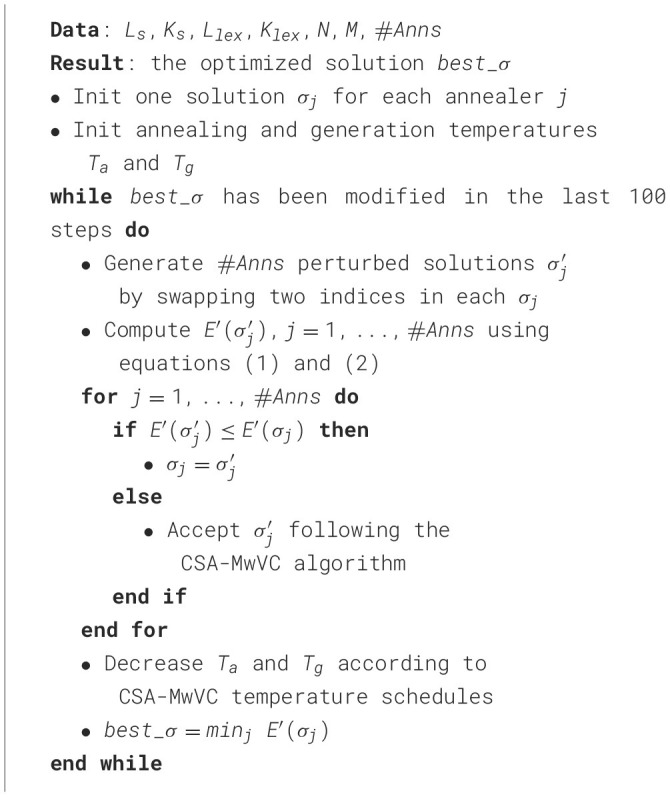
*CSA_OptMatcher*.

## 3 Experiments and results

In this section, we will detail the datasets employed to evaluate our method, along with the experiments conducted to simulate an automatic decipherment process. We utilize both established benchmarks introduced by prior works in this domain and novel datasets developed to contribute new benchmarks to the research community.

### 3.1 Datasets and corpora

Taking into account the introductory discussion, a practical decipherment problem may encompass various scenarios: matching Different Scripts used to write the Same Language (DS/SL), matching the Same Script for writing Different Languages (SS/DL), or, in the most challenging scenario, matching Different Scripts used to write Different Languages (DS/DL). Depending on the nature of the problem, two distinct mapping procedures need to be considered.

#### 3.1.1 Reference benchmarks from the literature

Some datasets have been employed in previous studies and have established themselves as standard benchmarks for assessing the effectiveness of computational tools designed to assist scholars in the decipherment process:

- **Ugaritic/Old Hebrew - U/OH**. Ugaritic, an ancient Semitic language closely related with Old Biblical Hebrew, was inscribed using a cuneiform script variant, while the latter employed the Hebrew alphabet. This scenario exemplifies a DS/DL problem category.
  Originally introduced by Snyder et al. (2010) for testing their system, the Ugaritic dataset has become a standard benchmark in the field. Consistent with Luo et al. (2019), our system evaluation involves two distinct settings: (a) Testing the model in a noiseless condition, akin to Berg-Kirkpatrick and Klein (2011), where only 2,214 cognate pairs are considered in both lexica. (b) Employing a slight modification of the Snyder et al. (2010) setting, which introduces a more challenging and realistic scenario by incorporating unpaired words in Ugaritic and Old Hebrew into the dataset (noisy setting). The original dataset comprised 7,267 Ugaritic and 39,635 Old Hebrew words, with only 2,214 forming cognate pairs. While the second setting may seem less realistic due to the abundance of data in both languages, it provides a valuable testbed. We reduced the number of non-cognate words, creating a dataset of 2,214 cognate words, 1,119 unpaired Ugaritic words, and 1,108 Old Hebrew words without corresponding cognates. These words were randomly selected from the dataset proposed in Snyder et al. (2010).
- **Linear B/Mycenaean Greek - LB/MG**. Linear B, a syllabic writing system employed for Mycenaean Greek dating back to approximately 1450 BC. Luo et al. (2019) curated a dataset by extracting pairs of Linear B and Greek words from a compiled lexicon, eliminating some ambiguous translations and resulting in 919 cognate pairs. This dataset holds particular significance for us, aligning with our emphasis on syllabic scripts from the Aegean region.
  For Linear B, the signs inventory is defined as the original set of signs in Linear B. Regarding Greek, given the syllabic nature of the mapping, complex signs comprised of all open syllables (excluding those indicating vowel quantity, such as syllables ending in η or ω) were included to streamline the signs inventory dimension on the Greek side.
  This dataset exemplifies a DS/SL problem category.
  The same authors introduced an additional, more challenging and noisy benchmark, presenting a scenario more realistic from a paleographic perspective. This benchmark uses the same Linear B lexicon but compares it with a pared-down Greek lexicon containing only proper nouns (LB/MG-names).

#### 3.1.2 New datasets

Considering our principal focus, it appeared justified to introduce novel datasets to serve as benchmarks for the decipherment of scripts/languages originating from the same expansive geographical region (East Mediterranean) and existing during a parallel timeframe (Bronze Age and early Iron Age).

- **Cypriot Syllabary/Arcadocypriot Greek - CS/AG**. The Cypriot Syllabary, a syllabic script employed in Iron Age Cyprus, features a right-to-left writing system. It evolved from the Cypro-Minoan syllabary, itself stemming from Linear A. Predominantly used in the Arcadocypriot dialect of Greek, this script provides another instance of a DS/SL problem.
  To construct a new dataset, we referred to the alphabetic-syllabic index in Hintze (1993). This dataset comprises 693 pairs of cognates, with the first written in the Cypriot Syllabary and the second in the Greek alphabet. Similar to the procedure applied in Luo et al. (2019) for the LB/MG dataset, any diacritics were removed from the Greek alphabet. Additionally, for Greek, only open syllables were considered, consistent with the approach used in the previous benchmark.
- **Phoenician/Ugaritic - Ph/Ug**. Phoenician, an extinct language originating from the Late Bronze Age region around Tire and Sidon, belongs to the Northwest Semitic language family. It exhibits notable similarities with Old Hebrew, Ugaritic, and other languages within the same linguistic group.
  To create a benchmark, we utilized the Semitic etymology database from StarlingDB, compiled by Alexander Militarev^6^. This online resource offers cognates for various Semitic languages, connecting them to Proto-Semitic forms. The resulting benchmark comprises 105 cognates and 58 unpaired words, encompassing both Phoenician and Ugaritic.
  This dataset exemplifies a DS/DL problem, akin to the U/OH dataset introduced earlier, as the two languages were written using distinct alphabetic scripts — the Phoenician alphabet for Phoenician and the Ugaritic cuneiform for Ugaritic.
- **Luvian/Hittite - Luv/Hit**. Hittite, an extinct Indo-European language spoken by the Hittites — a prominent Bronze Age Anatolian civilization — flourished in the 17th to 13th centuries BC. Primarily inscribed in a variant of cuneiform, distinct from the version used in Old Babylonian/Assyrian texts, Hittite played a crucial role in the expansive Hittite Empire.
  Luvian (or Luwian), another ancient language from the Anatolian branch of the Indo-European family, was contemporaneous with Hittite in Anatolia. Luvian manifested in two varieties: one employing cuneiform and the other using hieroglyphs. However, for this study, our focus remains on the cuneiform-based variant.
  Drawing on the meticulous work of Ringe et al. (2002), who compiled an etymologically verified wordlist featuring cognates across diverse Indo-European languages, we constructed a novel benchmark. This dataset encompasses 60 pairs of cognates between the two languages, coupled with 75 unpaired words introduced as “noise”.
  Notably, this dataset serves as a unique illustration of an SS/DL problem.

### 3.2 Evaluation

Regarding evaluation, we adhere to the methodology established in prior literature, notably following the approach delineated by Luo et al. (2019). Our evaluation focuses on quantifying the system's accuracy in identifying pairs of lost and known cognates enumerated in the provided dataset.

Emphasizing the guidance of Reimers and Gurevych (2017), who underscore the potential impact of system random initialization on results, we advocate reporting not only a single score but also the mean and standard deviation from multiple runs under the same configuration. This practice ensures a more nuanced understanding of the system's actual performance and facilitates robust comparisons. Consequently, the results presented in this paper include the mean and standard deviation of system accuracy across four runs, each initiated with distinct randomisations. This approach offers a comprehensive portrayal of our system's performance.

For consistency and comprehensive comparison with the system establishing state-of-the-art results, we replicated the experiments using the tool presented in Luo et al. (2019) on all the proposed datasets, adhering to the same experimental protocol we applied to evaluate our proposal. Moreover, we slightly modified their code to remove any information from the input not available in real decipherment settings, like the expected number of cognates in a given benchmark dataset.

### 3.3 Results

The two parameters, *N* and *M* associated with the solution shaping outlined earlier, may be viewed as hyperparameters for the proposed method, introducing more flexibility to potential solutions at the cost of additional parameters and potentially slower convergence. By increasing *N* or *M* the system gains the ability to incorporate intricate 1-to-many, many-to-1, or many-to-many mappings between the two sign inventories, offering versatility in specific scenarios. In our experiments, we chose to refrain from optimizing these parameters and adopted a straightforward rule: *N* = 1, *M* = 2 if |*L*_*s*_|>|*K*_*s*_| and *N* = 2, *M* = 1 otherwise.

To strongly discourage potentially degenerate solutions we set, in general, λ = 4 and λ = 8 for the “U/OH noisy” benchmark to further penalize inappropriate solutions.

Table 3 displays the outcomes of our experiments in comparison with the reference literature, specifically the system introduced in Berg-Kirkpatrick and Klein (2011) “*Matcher*” and in Luo et al. (2019) “*NeuroCipher*”.

**Table 3:** Accuracy results in cognate identification of *CSA_OptMatcher* compared to the reference literature.

	**Benchmark datasets**						

	**DS/DL**			**DS/SL**			**SS/DL**
	**U/OH**	**U/OH**	**Ph/Ug**	**LB/MG**	**LB/MG**	**CS/AG**	**Luv/Hit**
**System**	**Noiseless**	**Noisy**			**Names**		
*Matcher*	90.4	-	-	-	-	-	-
*NeuroCipher*	93.5	65.9*	-	84.7	67.3	-	-
*NeuroCipher* ^†^	90.4 ±0.64	**87.6** ±0.52	71.2 ±2.50	75.8 ±0.85	67.9 ±1.13	75.9 ±0.56	18.2^‡^ ±2.13
	(90.8)	(**88.26**)	(73.3)	(76.4)	(69.5)	(76.5)	(20.3)
*CSA_OptMatcher*	**95.5** ±0.83	74.7 ±1.26	**80.5** ±1.82	**89.4** ±1.81	**83.4** ±2.50	**86.3** ±1.73	**47.5** ±1.67
	(**96.3**)	(75.8)	(**82.9**)	(**91.0**)	(**87.0**)	(**87.9**)	(**48.3**)

In boldface the best result for each dataset. ^*^The comparison with the results in Luo et al. (2019) for the “U/OH noisy” dataset may not be entirely fair due to the use of a different, larger dataset. Inside round parentheses, the maximum Accuracy value obtained in our experiments is indicated. ^†^ Results for *NeuroCipher* computed or recomputed by us simulating a real setting and using the code in Luo et al. (2019). ^‡^ To enable the system to converge toward meaningful results we had to provide the number of cognates in the dataset, information not available in real settings.

Our system exhibits superior accuracy compared to any other work across almost all benchmark datasets, with a substantial margin. It is noteworthy that our results are presented as the mean and standard deviation of multiple runs, providing a more comprehensive assessment than the maximum accuracy achieved by the system, further highlighting the significance of the results. The only exception regards the U/OH noisy dataset for which a very large set of words is provided, a setting that clearly advantage methods based on Deep Neural Networks. However, this abundance of lexical items is not representative of real decipherment problems, which typically involve a few 100 words in each language, and it has been considered only for comparison purposes with past studies. On the contrary, we were not able to reproduce the results presented in Luo et al. (2019), leaving the impression that the reported Accuracies represent the maximum values obtained after numerous restarts. In real settings we cannot have the gold standard decipherment and restarting the tested method to maximize its performance is not a viable approach.

The datasets incorporating noise, such as U/OH noisy, Ph/Ug, LB/MG names, and Luv/Hit, present the most challenging scenarios among the seven benchmarks investigated in this study. The accuracy in identifying cognate words for these benchmarks is lower compared to other cases, but it remains remarkably high. Even in these more difficult scenarios, the system demonstrates an ability to automatically identify more than 50% of cognates in the two lexica. Such high accuracy would undoubtedly significantly enhance the contribution of this automated system to any paleographer's decipherment efforts.

## 4 Discussion and conclusions

We introduced a novel method for ancient scripts decipherment demonstrating its ability to yield excellent results in cognate identification compared to the current state-of-the-art. None of the hyperparameters were optimized, and it appears plausible that further improvements can be achieved by increasing the values of *N* and/or *M*. Our intention is to conduct additional experiments in this direction.

Another noteworthy aspect of the system pertains to its capability to consistently converge to reasonable solutions in any simulation. Throughout the development phase, the proposed system avoided being trapped into highly suboptimal solutions. While the simulations required a significant amount of time to converge, there was no necessity to restart the process, a practice commonly employed in methods of this nature [refer to, for instance, Berg-Kirkpatrick and Klein (2013)]. This confirms the efficacy of CSA as a function optimisation technique.

When replicating the experiments outlined in Luo et al. (2019) for the *NeuroDecipher* system, that defined the state of the art, and excluding all information about cognancy from the input, we observed significant differences. Overall, the Accuracies on the various datasets were slightly lower than reported in the original paper. Achieving convergence necessitated restarting the experiments multiple times using various random seeds, a practice impractical in real-world scenarios where a gold standard for comparison is absent.

There exist alternative approaches in the literature that we haven't explicitly addressed because they are not specifically designed for deciphering ancient scripts. However, these approaches tackle the challenge of deciphering substitution or homophonic codes, such as the well-known Zodiac-408 cipher or the Beale cipher (e.g., Ravi and Knight, 2011; Nuhn et al., 2013, 2014; Lasry et al., 2021, 2023). Ravi and Knight (2011) introduced a stochastic model incorporating both token n-grams and dictionaries. In cases where the target language is known, they can estimate a language model (LM) using a substantial dataset, even if artificially generated. This approach leverages complete lexica and frequency information specific to the known language. Unfortunately, applying these methods to decipher ancient languages poses challenges as the target language is often uncertain. It might be a language from the same region, sharing data scarcity similar to the lost language, making it impractical to construct useful LMs or rely on a comprehensive dictionary. In such scenarios, everything is only partially known or unreliable, including phonetic values, sign mappings, frequency information, and the true underlying language. These factors make it exceedingly challenging to apply methods like the one proposed by these authors.

Very interestingly, Lasry et al. (2021, 2023), even if working on a slightly different problem, successfully apply techniques similar to those proposed in this paper to decipher papal ciphers from the 16th to the 18th Century and Mary Stuart's lost letters from 1578-1584. They configured the problem as a combinatorial optimisation task and solved it by applying simulated annealing methods for exploring the search space in an efficient way.

While our automatic decipherment of ancient scripts has shown great promise, it would be misleading to infer that these tools can effortlessly resolve all outstanding issues in palaeography, epigraphy, and linguistics that have been debated by experts over the years. Despite their potential, these techniques encounter numerous challenges when applied in real decipherment scenarios: (a) The need for segmented and clean corpora is paramount. Constructing a corpus for an ancient undeciphered script, even after addressing segmentation problems and collecting single sign images and sign/word sequences, is a formidable task. Many inscriptions are damaged, with numerous signs being unreadable, and occurrences of broken words and partial sentences are commonplace. (b) Access to an extensive cognate list is crucial, yet in most real cases, only two word lists are available for matching, without any assurance that cognates from the lost language truly exist in the lexicon of the known language. (c) In natural language processing (NLP), evaluations are typically conducted on well-established test beds and the studies discussed earlier focused on well-known correspondences to demonstrate system effectiveness. On the contrary, testing these systems on real cases involving unknown writing systems and their corresponding languages presents an entirely different set of challenges and uncertain comparanda.

Considering these factors, we concur with Sproat (2020), who proposed that these tools can assist paleographers in illuminating the decipherment process. However, we cannot solely depend on them to offer a comprehensive solution to our actual challenges without human intervention to guide the process and interpret the results. Nonetheless, the excellent performance of these tools in identifying the cognates can start the ‘domino effect' that precedes the decipherment by indicating to the paleographer some relevant correspondences that will indicate her/his path to success.

Our future endeavors involve applying the proposed system to undeciphered scripts from the Aegean area. We aim to contribute insights that may finally address longstanding problems unresolved for centuries.

## Data Availability

The datasets and codes presented in this study can be found in online repositories. The names of the repository/repositories and accession number(s) can be found at: https://github.com/ftamburin/CSA_OptMatcher.

## Footnotes

^1^Words with similar meanings in different languages that share an etymological ancestor in a common parent language.

^2^Here, “alphabet” denotes a broad concept of inventories of signs, glyphs, etc., constituting a writing system.

^3^The Generalized Edit Distance is a metric, its upper bound is 1 and it does not escalate repetitions remaining simple and quick to calculate.

^4^ https://github.com/ftamburin/EditDistanceWild

^5^ https://github.com/structurely/csa

^6^The Tower of Babel, https://starlingdb.org.
